# Boyle’s Law ignores dynamic processes in governing barotrauma in fish

**DOI:** 10.1038/s41598-023-46125-9

**Published:** 2023-11-05

**Authors:** J. R. Kerr, P. R. White, T. G. Leighton, L. G. M. Silva, P. S. Kemp

**Affiliations:** 1 International Centre for Ecohydraulics Research, Faculty of Engineering and Physical Sciences, University of Southampton, Boldrewood Campus, Southampton, SO16 7QF UK; 2 Institute of Sound and Vibration Research, University of Southampton, Southampton, SO17 1BJ UK; 3 Stocker Lab, Institute for Environmental Engineering, Department of Civil, Environmental and Geomatic Engineering, ETH-Zurich, 8046 Zurich, Switzerland

**Keywords:** Animal migration, Freshwater ecology, Energy infrastructure, Applied physics

## Abstract

The expansion and potential rupture of the swim bladder due to rapid decompression, a major cause of barotrauma injury in fish that pass through turbines and pumps, is generally assumed to be governed by Boyle’s Law. In this study, two swim bladder expansion models are presented and tested in silico. One based on the quasi-static Boyle’s Law, and a Modified Rayleigh Plesset Model (MRPM), which includes both inertial and pressure functions and was parametrised to be representative of a fish swim bladder. The two models were tested using a range of: (1) simulated and (2) empirically derived pressure profiles. Our results highlight a range of conditions where the Boyle’s Law model (BLM) is inappropriate for predicting swim bladder size in response to pressure change and that these conditions occur in situ, indicating that this is an applied and not just theoretical issue. Specifically, these conditions include any one, or any combination, of the following factors: (1) when rate of pressure change is anything but very slow compared to the resonant frequency of the swim bladder; (2) when the nadir pressure is near or at absolute zero; and (3) when a fish experiences liquid tensions (i.e. negative absolute pressures). Under each of these conditions, the MRPM is more appropriate tool for predicting swim bladder size in response to pressure change and hence it is a better model for quantifying barotrauma in fish.

## Introduction

Globally, the development of water infrastructure is expanding at evermore rapid rates to meet the needs of a growing population, manifestly modifying freshwater ecosystems^1^. The development of water infrastructure, for purposes such as power generation, water level management and flood defence, is considered one of the greatest threats to the conservation of freshwater ecosystems^2^, posing substantial challenges for sustainable development. For fish that move between critical habitats fragmented by river infrastructure, injury and mortality is common among those that transit via hazardous routes, with one in five (22.3%) that pass through hydropower turbines killed^3^ and similar or higher levels frequently reported for pumps^4,5^. Barotrauma from exposure to abrupt pressure change is thought to be a key contributing factor to turbine and pump passage mortality^6,7^. Despite pioneering research undertaken in the Pacific Northwest^8–10^ and elsewhere^11–13^ that highlights barotrauma as being an important pathway for injury of a range of fish species during turbine passage, there remain many uncertainties regarding its causal mechanisms.

Barotrauma is conventionally assumed to be the result of two physical processes. First, the expansion of undissolved gas due to rapid reduction in pressure may cause the swim bladder to rupture^9^, possibly resulting in secondary trauma as gas escapes and damages the surrounding tissues and other organs^8^. The correlation between pressure change and gas expansion frequently stated as governing this relationship is that described by Boyle’s Law, which is isothermal:

1 $${P}_{1}{V}_{1}= {P}_{2}{V}_{2},$$

where $P$ and $V$ are pressure and volume, respectively, at time $1$ and $2$. The second physical process relates to dissolved gas within the body fluids coming out of solution and penetrating and damaging tissues^9^. The correlation frequently noted as governing this relationship is that described by Henry’s Law, which states that the concentration of a dissolved gas is directly proportional to the partial pressure of that gas above the solution. It is thought that most injuries caused by rapid decompression are a result of the expansion of undissolved gas, which is conventionally assumed to be governed by Boyle’s Law^9^.

Both Henry’s and Boyle’s Law are simplistic in that they express only the equilibrium states. Gas expansion and dissolution estimates using these laws will be erroneous if dynamic phenomenon contribute in a meaningful way to volume changes and gas flux in the periods between the initial and final conditions. For example, a gas bubble in a liquid exposed to rapid decompression can act dynamically and an excess of kinetic energy can cause it to overshoot its equilibrium state, expanding to a volume beyond that predicted by Boyle’s Law^14^. As it does so, the kinetic energy is converted to potential energy and the expansion slows and eventually stops, until the bubble begins to contract again and starts to oscillate about its equilibrium state^15^. This oscillation is an inevitable consequence of the inertia of the liquid in a lightly damped system^16^. This process is analogous to suddenly adding weight to the bottom end of a vertically suspended coiled spring, which does not instantly transform from being motionless at its start to motionless at its end position (the behaviours analogous to the Boyle’s Law assumptions), but typically overshoots and oscillates about its final equilibrium state. As such, under some conditions, a swim bladder may not only expand to a much greater size but also contract to a much smaller size than predicted by Boyle’s Law, which assumes equilibrium occurs instantaneously. Large amplitude oscillations and greater swim bladder volume may increase the risk of rupture, especially if the expansion is beyond the elastic limits of critical tissues. In addition, contraction of the swim bladder to a smaller size than normally occurs during buoyancy regulation (the primary function of the swim bladder) may also have deleterious effects^17^. Rate of decompression is a key factor that will influence, along with the presence of negative absolute pressures (i.e. tension), the probability of such overexpansion and contraction occurring^14^.

A model based on the Rayleigh Plesset equation^18,19^ which dynamically incorporates both inertial and pressure functions, is likely to be a better predictor of the expansion or contraction of a gas bubble exposed to rapid pressure change than that derived by Boyle’s Law alone. The Rayleigh Plesset equation is the most commonly used nonlinear equation of motion for predicting the damped pulsation of a spherical gas bubble in an infinite body of liquid^18^. In comparison to Boyle’s Law, which does not account for inertia or damping, it is able to predict bubble overshoot, rebound and oscillation, which are to be expected in any lightly damped system when rate of pressure change is anything but very slow compared to the resonant frequency of the bubble. The Rayleigh Plesset equation also produces realistic outputs when the absolute pressure is near or below zero (and indeed for those intervals when the liquid is in tension)^20,21^. In comparison, when the external pressure becomes zero, Boyle’s Law predicts that the gas volume increases without bounds. This is unrealistic as the rate of gas expansion into an infinitely large space would take time to occur. In addition, when the external absolute pressure is negative, Boyle’s Law is inappropriate as it predicts negative volumes of gas. This does not render Boyle’s Law invalid, as the most parsimonious model should always be used if it produces useful results for the range of conditions assessed. However, rapid rates of pressure change can occur during passage through water infrastructure^22^ and very low pressures and even tension can occur near turbine^23^ and pump^24^ blades so it is important to assess whether Boyle’s Law is appropriate as a base model for predicting swim bladder expansion given the range of conditions to which a fish may be exposed.

In this study we present a Modified Rayleigh Plesset Model (MRPM) parameterised so that it is representative of a fish swim bladder. We compare swim bladder size predicted by the MRPM and Boyle’s Law under a range of: (1) simulated pressure profiles, and (2) empirically derived pressure profiles recorded at water infrastructure worldwide. As the Rayleigh Plesset equation incorporates both inertia and pressure functions and can accurately predict the expansion or contraction of a gas bubble exposed to rapid pressure change, even when absolute pressure is near or below zero, we consider the MRPM to be the more realistic model and compare swim bladder size predicted by Boyle’s Law to it. We aim to: (a) highlight conditions where Boyle’s Law is likely erroneous in predicting swim bladder size in response to pressure change, and (b) assess whether such conditions occur in situ, respectively. The findings of this study provide a critical step forward in refining understanding of the physical processes leading to barotrauma in fish.

## Methods

### Swim bladder expansion models

Two swim bladder expansion models, one based on the Rayleigh Plesset equation and one on Boyle’s Law, were formulated in Matlab 2017a (MathWorks). Both models assumed a spherical swim bladder and predicted swim bladder volume in response to pressure change. Differences in maximum and minimum volume predicted by each model were compared for: (1) a range of simulated pressure profiles, and (2) empirically derived pressure profiles (existing data) recorded at three hydropower dams worldwide and a pumping station in Belgium. Absolute pressures were used in the models and are reported accordingly.

#### Boyle’s Law model (BLM)

For this model, a generalisation of Boyle’s Law was used that includes both heat and work transfer (i.e. it is polytropic):

2 $${V}_{2}={{V}_{1}\left(\frac{{P}_{1}}{{P}_{2}}\right)}^{1/\kappa }.$$

where κ is the polytropic index, an engineering approximation that takes a value of unity when the gas changes are isothermal (i.e. Boyle’s Law), a value equal to the ratio of the specific heat (*ca.* 1.4) when the gas changes are adiabatic, and an intermediate value if the gas changes are neither adiabatic nor isothermal^14^. In this study, an intermediate value of *κ* = 1.2 is chosen because it is uncertain to what degree the process is isothermal or adiabatic. The polytropic index is used in the BLM to produce comparable results to the MRPM, for which κ is a key parameter. The BLM computes swim bladder volume at each time point based solely on the external pressure at that time instant.

#### Modified Rayleigh Plesset model (MRPM)

The Rayleigh Plesset equation (Eq. 3)^18,19^ is able to predict the spherical radius *R* of a gas bubble surrounded by an infinite body of incompressible liquid of density, $\rho$. It captures the dynamics of the process in a way that Boyle’s Law does not.

3 $$R\ddot{R}+{\frac{3\dot{R}}{2}}^{2}=\frac{1}{\rho }\left\{\left({P}_{0}+\frac{2\sigma }{{R}_{0}}-{p}_{\mathrm{v}}\right){\left(\frac{{R}_{0}}{R}\right)}^{3\kappa }+{p}_{\mathrm{v}}-\frac{2\sigma }{R}-\frac{4\eta \dot{R}}{R}-{P}_{0}-P\left(t\right)\right\},$$

where $\dot{R}= \partial R/\partial t$, $\ddot{R}= {\partial }^{2}R/\partial {t}^{2}$, ${P}_{0}$ is the pressure in the liquid outside the bubble, $\sigma$ is the surface tension of the bubble (causing the $2\sigma /R$ Laplace pressure term^14^), ${R}_{0}$ is the equilibrium bubble radius, ${p}_{\mathrm{v}}$ is the vapour pressure, $\eta$ is the shear viscosity of the liquid, and $P\left(t\right)$ is pressure in the liquid at time $t$. As in the BLM, in this model a polytropic index, $\upkappa$, equal to 1.2 is employed. To keep outputs between models comparable under equilibrium conditions and focus on the effects of dynamic processes, in this study, ${p}_{\mathrm{v}}$ is set to zero in the MRPM. The only dissipation mechanism in Eq. (3) is through shear viscosity. Radiation loss cannot be accounted for because of the assumption of an incompressible fluid, whilst to incorporate net thermal losses would require that the polytropic index change over time^25^. Equation (3) represents the gas pocket as a sphere at all times but if the actual gas body departs from sphericity this does not affect the predicted pressure greatly, because (i) the representation of Eq. (3) in terms of the equivalent radius the gas body would have if the same volume were spherical, can be mapped entirely into a nonlinear equation in terms of bubble volume^18^; and (ii) it is changes in volume, not shape, that determine the pressure in the gas body in conditions where that pressure is homogenous throughout the gas body volume^26^.

In this study, a modified version of the Rayleigh Plesset equation is used whereby the gas bubble is parameterised to be representative of a fish swim bladder. This was achieved by selecting appropriate $\eta$ and σ values in Eq. (3). The value of $\eta$ in the MRPM does not have a simple physical meaning representing combined physical properties of the bladder and associated tissue, however, it can be fitted to real-world measurements by consideration of the quality factor ($Q$), also known as a damping factor^27–29^. $Q$ is a dimensionless value that describes how damped an oscillator or resonator is, with $Q$>1 and $Q$<1 corresponding to underdamped and overdamped oscillators, respectively. Resonators with high $Q$ have low damping, so that they ring or vibrate longer. Through comparison of the linearised Rayleigh Plesset and standard mass spring damper system equations (see Supplementary Equations), a relationship between $\eta$ and $Q$ (Eq. 4) can be derived for use in Eq. (3):

4 $$Q= \frac{\sqrt{\rho }{R}_{0}\sqrt{3\kappa \left({P}_{0}-{p}_{v}\right)+\left(\frac{2\sigma }{{R}_{0}}\right)\left(3\kappa -1\right)-\frac{4{\eta }^{2}}{{{\rho R}_{0}}^{2}}}}{4\eta }.$$

For fish swim bladders, $Q$ varies depending on a range of factors including its volume and shape^30^ and the viscous properties of its wall and surrounding tissues^31^, and as such varies between species^28^. $Q$ also varies with depth as energy loss decreases at greater pressures^28^. Fish swim bladders are typically attributed $Q$ values of between 1 and 10 ($Q$ = 3.0–5.0^27^; 1.8–3.0^32^; 1.0–3.5^31^; 1.0–4.1^33^; 3–10^34^; 1.1–3.0^28^; 3.7–10^29^). In this study, differences in the minimum and maximum swim bladder volume predicted by the BLM and the MRPM were assessed at $Q$ = 1, 5 and 10 to cover the full range of possible values. To further parameterise the MRPM to be representative of a fish swim bladder, σ in Eq. (3) was set as 0.0055 N m^-1^, which is the estimated value for goldfish, *Carassius auratus*^34^ (Table 1). The MRPM (Eqs. 3 and 4) was solved in Matlab using a Runge–Kutta pair (4th and 5th order) and changes in volume, $V=\frac{4}{3}\pi {R}^{3}$, in response to pressure changes reported.

**Table 1:** Constant values used in the modified Rayleigh Plesset model (MRPM) to predict swim bladder radius in response to pressure change.

Parameter	Symbol	Value	Unit
Initial pressure in the liquid outside the bubble	${P}_{0}$	500,000	Pa
Vapour pressure	${p}_{\mathrm{v}}$	0	Pa
Swim bladder surface tension	σ	0.0055	N m^–1^
Initial bubble radius	${R}_{0}$	0.0156	m
Polytropic index	$\kappa$	1.2	Dimensionless
Water density	$\rho$	1000	kg m^–3^

### Simulated pressure profiles

**Figure 1:**
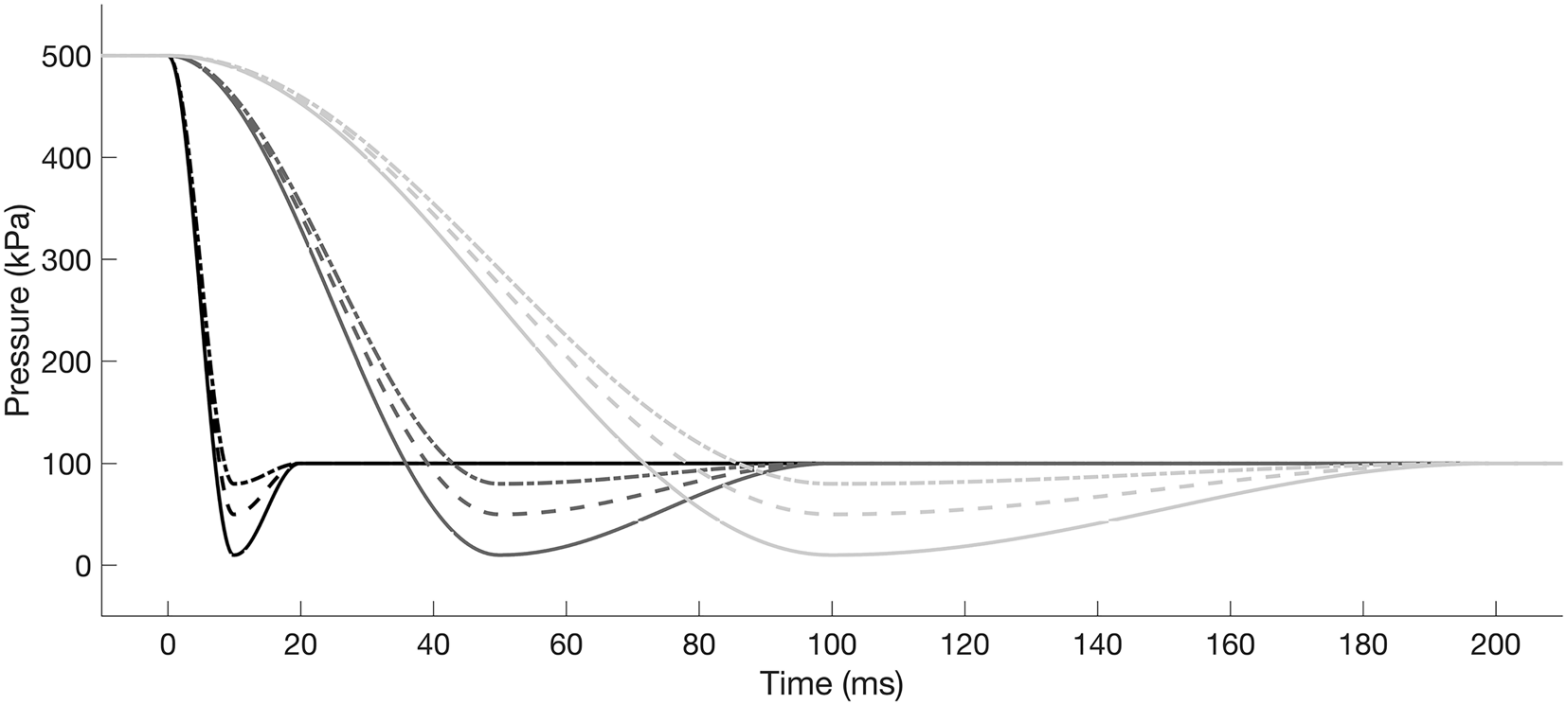
Examples of the simulated pressure profiles used to compare swim bladder volume predicted by the numerical models. Black, dark grey and light grey are decompression durations ($D$) of 10, 50 and 100 ms, respectively. Solid, dashed and dot-dashed lines are nadir pressures (${P}_{\mathrm{n}}$) of 10, 50 and 80 kPa, respectively.

The simulated pressure profiles (SPP) were a simplified version of the pressure changes experienced by a depth acclimated fish passing through a hydropower plant with a deep water intake^8^: (1) a high initial static pressure (${P}_{\mathrm{start}}$) relating to a fish residing in deep water, (2) a rapid drop to the nadir pressure (${P}_{\mathrm{n}}$) relating to a fish being entrained into the hydropower plant and passing the turbine blades, (3) a rapid recompression as the fish is expelled out through the draft tube, and 4) static ambient post-passage pressure equivalent to those experienced in the tailrace (${P}_{\mathrm{end}}$). The decompression and recompression components of the SPP (2 and 3, respectively) were simulated with a cosine function using Eq. (5).

5 $$\begin{aligned} P\left( t \right) & = P_{{{\text{start}}}} , &t < t_{{{\text{start}}}} \\ &= 0.5\left( {P_{{{\text{start}}}} - P_{{\text{n}}} } \right)cos\left( {\pi \left( {\frac{{t - t_{{{\text{start}}}} }}{{t_{{{\text{end}}}} - t_{{{\text{start}}}} }}} \right)} \right) + 0.5\left( {P_{{{\text{start}}}} - P_{{\text{n}}} } \right), &t_{{{\text{start}}}} < t < t_{{\text{n}}} \\ &= 0.5\left( {P_{{\text{n}}} - P_{{{\text{end}}}} } \right)\cos \left( {\pi \left( {\frac{{t - t_{{{\text{start}}}} }}{{t_{{{\text{end}}}} - t_{{{\text{start}}}} }}} \right)} \right) + 0.5\left( {P_{{\text{n}}} - P_{{{\text{end}}}} } \right), &t_{{\text{n}}} < t < t_{{{\text{end}}}} \\ &= P_{{{\text{end}}}} , &t_{{{\text{end}}}} < t \\ \end{aligned}$$

where ${t}_{\mathrm{start}}$ and ${t}_{\mathrm{end}}$ are the times at the start and end of the pressure change, respectively. In this study, the nadir time, ${t}_{\mathrm{n}}$, was considered to be midway between the start and end time, i.e. ${(t}_{\mathrm{start}}+{t}_{\mathrm{end}})/2$. Two key factors that are known to influence barotrauma in fish as they pass through a hydropower plant are the ratio of pressure change (RPC)^6^, which is the quotient of the acclimation and nadir pressures, and the rate of pressure change (${P}_{\mathrm{rate}}$)^9,11^. To resolve conditions that produce differences between the two swim bladder expansion models, a range of SPPs were generated with variable RPC and ${P}_{\mathrm{rate}}$, which was achieved by adjusting the nadir pressure (${P}_{\mathrm{n}}$) between − 20 and 100 kPa and the duration ($D$) of the decompression and compression components of the SPP between 1 and 1000 ms, respectively, while keeping ${P}_{\mathrm{start}}$ (500 kPa) and ${P}_{\mathrm{end}}$ (100 kPa) constant (Fig. 1).

### Empirical pressure profiles

**Figure 2:**
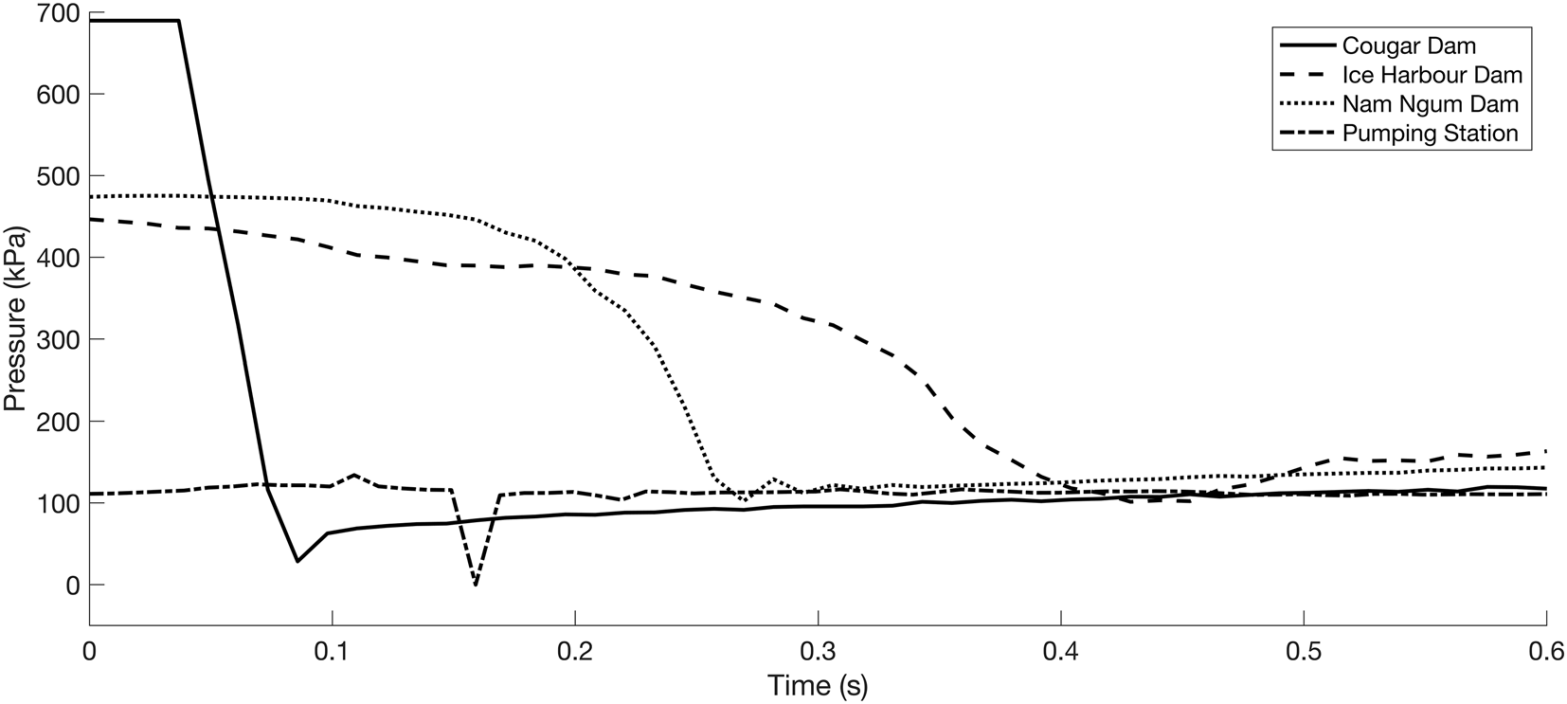
Decompression profiles from three hydropower dams (Cougar, Ice Harbour and Nan Ngum) and a pumping station (Duivelsput) used for the swim bladder expansion modelling.

Four pressure profiles (existing data) were obtained for three hydropower dams worldwide and a pumping station in Belgium (Table 2, Fig. 2). The data was collected using neutrally buoyant autonomous sensors (either sensor fish^35,36^ or Barotrauma detection system [BDS]^37^), which were released through the hydropower plants or pumping station to measure the pressures likely experienced by an organism during transit. The empirical pressure profiles used in this study were selected as they were most likely to produce differences in the outputs between the MRPM and the BLM, i.e. the pressure profiles exhibited either: (1) rapid rates of change, or (2) contained nadir pressures near or below absolute zero. At the Duivelsput pumping station (Table 2; Fig. 2), a transient (one record, sample rate: 100 Hz) and very small negative absolute pressure (− 1.73 × 10^–34^ Pa), not meaningfully different from zero, were recorded during the sensors passage through the facility. Its transient nature casts doubt on its authenticity. However several lines of evidence support using the dataset as part of this study: (1) similar negative absolute pressures were recorded simultaneously across all three of the probe’s pressure sensors, (2) the transient low pressure event occurred during a transitional phase when data from other sensors in the probe (e.g. accelerometers) suggest it was passing through the pump (Fig. S1) and (3) similar pressure profiles were recorded at the same pumping station using identical sensors but with slightly higher nadir values (i.e. no tension) (Fig. S2). Importantly, near zero and negative absolute pressures do occur within pumps, as evidenced by numerical modelling^24^ and cavitation damage that occurs on the blades^38^. Hence, even if the Duivelsput dataset is of questionable accuracy in terms and lacking extensive replication tests, it is not unrealistic.

**Table 2:** Metadata for the turbine and pump passage pressure profiles, collected using autonomous sensors, used within the swim bladder gas expansion model.

Dam	Location	Dam height (m)	Turbine/pump type	Turbine/pump capacity	Operational conditions during data collection				Sensor	Data source
					Release description	$h$(m)	$d$(m^3^ s^–1^)	Sensor release depth (m)		
Ice Harbor Dam	Snake River, USA	31	Kaplan	3 × 90 MW; 3 × 111 MW	Unit 1 (90 MW turbine), upper 1% of peak efficiency, deep release	30.2	402	*ca.* 38	Gen 2 Sensor Fish^36^	^22^
Nam Ngum Dam	Nam Ngum River, Laos	70	Francis	2 × 17.5 MW; 3 × 40 MW	Unit 1 (17.5 MW turbine)	38.7	52	14	Gen 2 Sensor Fish^36^	^39^
Cougar Dam	McKenzie River, USA	138	Francis	2 × 12.5 MW	Unit 2, maximum wicket gate opening	88.1	16	Penstock entrance in the dam forebay	Gen 1 Sensor Fish^35^	^40^
Duivelsput pumping station	Oude Kale River, Belgium	1.87	Axial flow [AF] and Fairbanks Nijhuis [FN] pump	AF: 3 × 1 m^3^ s^–1^; FN: 2 × 1.1 m^3^ s^–1^	FN pump no.1 at 40 Hz (~ 468 rpm)	1.87	0.76	Water surface	BDS sensor^37^	*Pers. Comm.* Jeffrey Tuhtan

$h$ hydraulic head, $d$ turbine/pump discharge.

### Comparison of model outputs

Two metrics were used to compare the outputs of the models, the quotient of the: (1) maximum (${v}_{\mathrm{max}}$: Eq. (6)), and (2) minimum (${v}_{\mathrm{min}}$: Eq. (7)) swim bladder volume predicted by each model:

6 $${v}_{\mathrm{max}}={V}_{\mathrm{max}}^{\mathrm{BLM}}/{V}_{\mathrm{max}}^{\mathrm{MRPM}},$$

7 $${v}_{\mathrm{min}}={V}_{\mathrm{min}}^{\mathrm{MRPM}}/{V}_{\mathrm{min}}^{\mathrm{BLM}},$$

where ${V}_{\mathrm{min}}^{\mathrm{BLM}}$ and ${V}_{\mathrm{max}}^{\mathrm{BLM}}$ are the minimum and maximum predicted swim bladder volume for the BLM, respectively, with ${V}_{\mathrm{min}}^{\mathrm{MRPM}}$ and ${V}_{\mathrm{max}}^{\mathrm{MRPM}}$ representing the equivalent for the MRPM. A value of ${v}_{\mathrm{max}}$ less than one indicates that the BLM underestimates the maximum swim bladder volume compared to the MRPM (i.e. the BLM predicted expansion to be less extreme). Values of ${v}_{\mathrm{min}}$ less than one equate to the BLM predicting a larger minimum swim bladder volume compared to the MRPM (i.e. the BLM predicted contraction to be less extreme). Data is lacking on the threshold at which ${v}_{\mathrm{max}}$ or ${v}_{\mathrm{min}}$ is biologically significant, i.e. has individual fitness costs (see “Discussion”). In this study, a difference of > 5% (i.e. $v$ > 1.05 or $v$ < 0.95) is conservatively used as a cut-off to highlight potentially important differences in model outputs. For the SPPs, to highlight situations where differences between the models occurred, ${v}_{\mathrm{max}}$ and ${v}_{\mathrm{min}}$ were calculated, and the results visualised with 2-dimensional surface plots with nadir pressure on the x-axis and duration on the y-axis. Results are presented for only the SPPs with positive nadir values as the BLM does not produce physical outputs under negative pressures and hence ${v}_{\mathrm{max}}$ and ${v}_{\mathrm{min}}$ values are not meaningful. Alongside the surface plots, representative examples of the SPPs that caused ${v}_{\mathrm{max}}$ and ${v}_{\mathrm{min}}$ to differ significantly from unity and the predicted swim bladder volume for each model are provided. Representative examples are provided at $Q$ = 5 as observed trends in swim bladder size in response to rapid decompression at this value were representative of those at 1 and 10, with only the relative difference in the magnitude of ${v}_{\mathrm{max}}$ and ${v}_{\mathrm{min}}$ varying between the different quality factors tested. For the empirical pressure profiles, the swim bladder volume predicted by each model are presented and the ${v}_{\mathrm{max}}$ and ${v}_{\mathrm{min}}$ values stated. For the empirical profiles the internal swim bladder pressure predicted by the MRPM is also shown. For the BLM, the internal pressure is equal to the external driving pressure (i.e. the empirical pressure profiles) so this isn’t shown.

## Results

### Simulated pressure profiles

**Figure 3:**
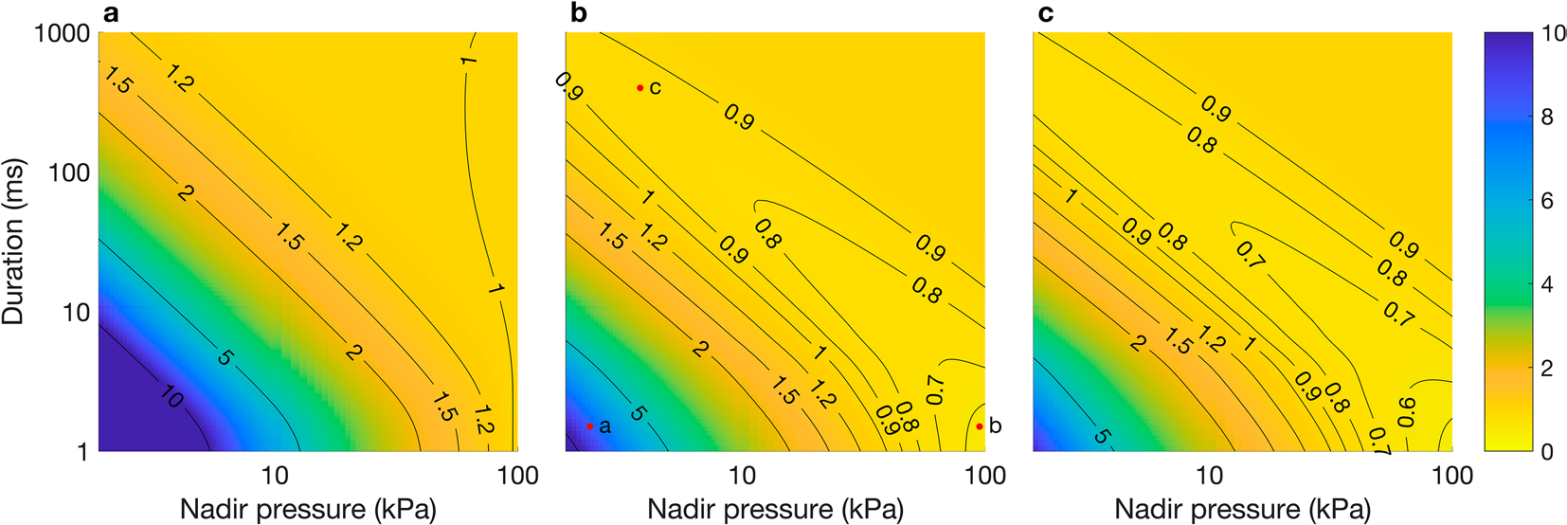
Surface plots of ${v}_{\mathrm{max}}$, the quotient of the maximum swim bladder volume predicted by the BLM and MRPM in response to decompression for a range of nadir pressures (${P}_{\mathrm{n}}$: kPa) and durations ($D$: ms) with a Quality Factor ($Q$) of 1 (**a**), 5 (**b**) and 10 (**c**). Within subplot (**b**), red dots labelled ‘a’ to ‘c’ are points for which the swim bladder volume predicted by each model are shown in Fig. 4a–c, respectively.

At all $Q$ values tested, the BLM overestimated the maximum swim bladder volume compared to the MRPM (${v}_{\mathrm{max}}$ > 1) when decompression occurred rapidly and the nadir pressure was low (e.g. $D$ = 1 ms, ${P}_{\mathrm{n}}$ = 1 kPa) (Fig. 3a–c). The magnitude by which the BLM overestimated the maximum swim bladder volume compared to the MRPM (${v}_{\mathrm{max}}$ > 1) was greater at $Q$ = 1 than 5 or 10 (Fig. 3a–c). At $Q$ = 5 and 10 the BLM underestimated the maximum swim bladder volume compared to the MRPM (${v}_{\mathrm{max}}$ < 1) when decompression occurred rapidly and the nadir pressure was high (e.g. $D$ = 1 ms, ${P}_{\mathrm{n}}$ = 100 kPa) (Fig. 3b,c). At $Q$ = 5 and 10, the BLM also slightly underestimated the maximum swim bladder volume compared to the MRPM (${v}_{\mathrm{max}}$ < 1) when decompression was slow and the nadir pressure was low (e.g. $D$ = 1000 ms, ${P}_{\mathrm{n}}$ = 1 kPa) (Fig. 3b,c). The magnitude by which the BLM underestimated the maximum swim bladder volume compared to the MRPM was slightly greater at $Q$ = 10 than 5, evidenced by the slightly lower ${v}_{\mathrm{max}}$ values in the $Q$ = 10 surface plot (Fig. 3b,c). The BLM did not underestimate maximum swim bladder size at $Q$ = 1.

**Figure 4:**
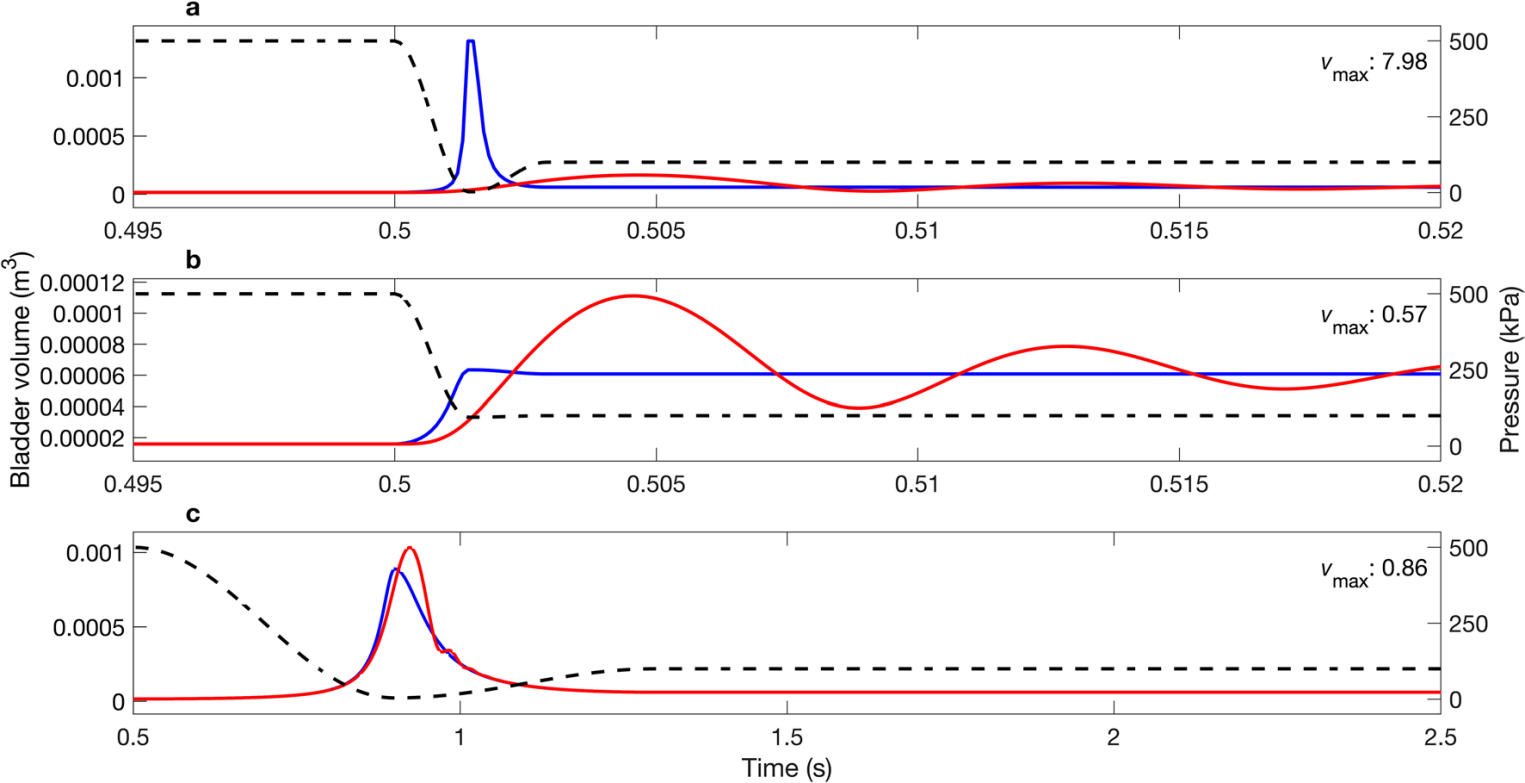
Examples of simulated pressure profiles (right axis: dashed black line) for which the maximum swim bladder volume (m^3^; left axis) predicted by the BLM (blue line) and MRPM (red line) differed: (**a**) $D$= 1.5 ms, ${P}_{\mathrm{n}}$ = 2.5 kPa, (**b**) $D$ = 1.5 ms, ${P}_{\mathrm{n}}$ = 95 kPa, (**c**) $D$= 400 ms, ${P}_{\mathrm{n}}$ = 4 kPa. MRPM data for $Q$ = 5. Quotient of maximum swim bladder volume (${v}_{\mathrm{max}}$) predicted by each model inset in top right of each panel. See Fig. 3b for corresponding location of each example within the parameter space.

Representative examples of pressure profiles that caused ${v}_{\mathrm{max}}$ to deviate from unity (Fig. 4) include those where: (1) decompression occurred rapidly ($D$ = 1.5 ms) and the nadir pressure was very low (${P}_{\mathrm{n}}$ = 2.5 kPa) causing the BLM to overestimate maximum swim bladder size (${v}_{\mathrm{max}}$ = 7.98) (Fig. 4a); (2) decompression occurred rapidly ($D$ = 1.5 ms) but the nadir pressure was high (${P}_{\mathrm{n}}$ = 95 kPa) causing the BLM to underestimate maximum swim bladder size (${v}_{\mathrm{max}}$ = 0.57) (Fig. 4b); and (3) decompression occurred slowly ($D$ = 400 ms) and the nadir pressure was very low (${P}_{\mathrm{n}}$ = 4 kPa) causing the BLM to underestimate maximum swim bladder size (${v}_{\mathrm{max}}$ = 0.86) (Fig. 4c).

**Figure 5:**
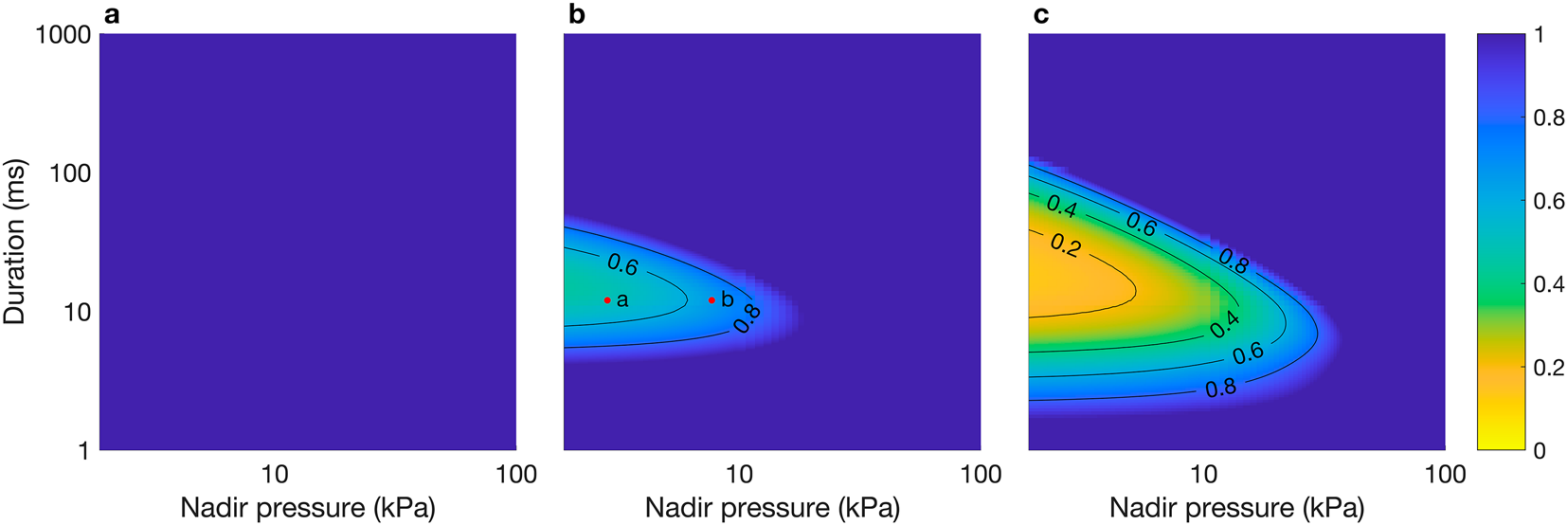
Surface plots of ${v}_{\mathrm{min}}$, the quotient of the minimum swim bladder volume predicted by the MRPM and BLM over a range of nadir pressures (${P}_{\mathrm{n}}$: kPa) and durations ($D$: ms) for the Simulated Pressure Profiles (SPP) for $Q$ =1 (**a**), 5 (**b**) and 10 (**c**). Within subplot (**b**), red dots labelled ‘a’ and ‘b’ are points for which the swim bladder volume predicted by each model are shown in Fig. 6a and b, respectively.

At $Q$ = 1, there was no difference in the minimum swim bladder volume predicted by the BLM and the MRPM (Fig. 5a). At $Q$ = 5 and 10, the BLM underestimated the minimum swim bladder volume compared to the MRPM (${v}_{\mathrm{min}}$ < 1) under a range of conditions; mostly when decompression occurred reasonably quickly (*ca.* $D$= 2–130 ms) and nadir pressures were low (*ca.* ${P}_{\mathrm{n}}$ < 35 kPa) (Fig. 5b,c). The greatest underestimation (${v}_{\mathrm{min}}$ < 0.2) occurred at $Q$ = 10, at very low nadir pressures (e.g. *ca.* ${P}_{\mathrm{n}}$ < 6 kPa) and when decompression duration was relatively quick (ca. $D$ = 10–40 ms) (Fig. 5c).

**Figure 6:**
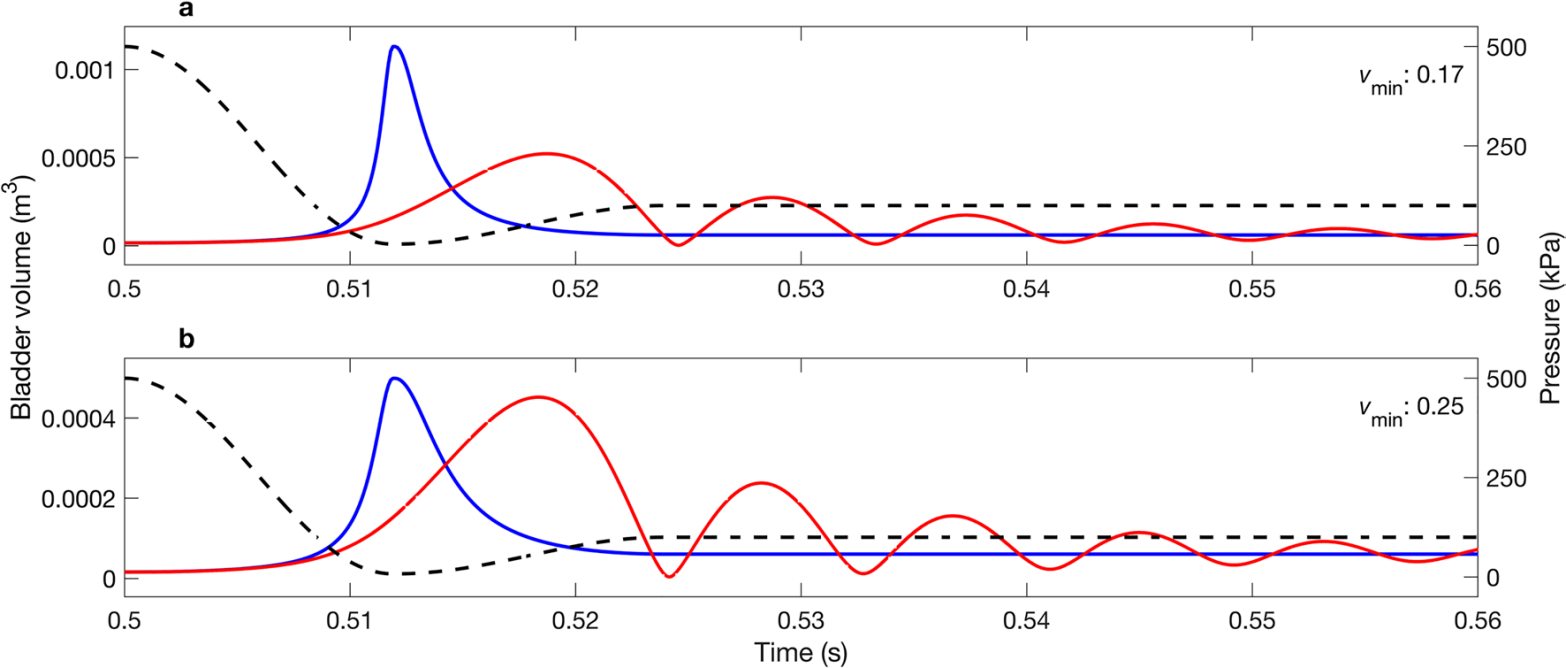
Examples of simulated pressure profiles (kPa; right axis: dashed black line) for which the minimum swim bladder volume (m^3^; left axis) predicted by the BLM (blue line) and the MRPM (red line) differed: (**a**) $D$ = 12 ms, ${P}_{\mathrm{n}}$ = 3 kPa and (**b**) $D$ = 12 ms, ${P}_{\mathrm{n}}$ = 8 kPa. MRPM data for $Q$ = 5. Quotient of minimum swim bladder volume (${v}_{\mathrm{min}}$) predicted by each model inset in top right of each panel. See Fig. 5b for corresponding location of each example within the parameter space.

Representative examples of pressure profiles that caused ${v}_{\mathrm{min}}$ to deviate from unity are shown in Fig. 6. Note that similar trends occurred for each example presented, but that the amplitude of the contraction that occurred as part of the first oscillation is greater (i.e. a smaller swim bladder size is predicted) for pressure profiles with lower nadir pressures (e.g. $D$ = 12 ms, ${P}_{\mathrm{n}}$ = 3 kPa: Fig. 6a).

**Figure 7:**
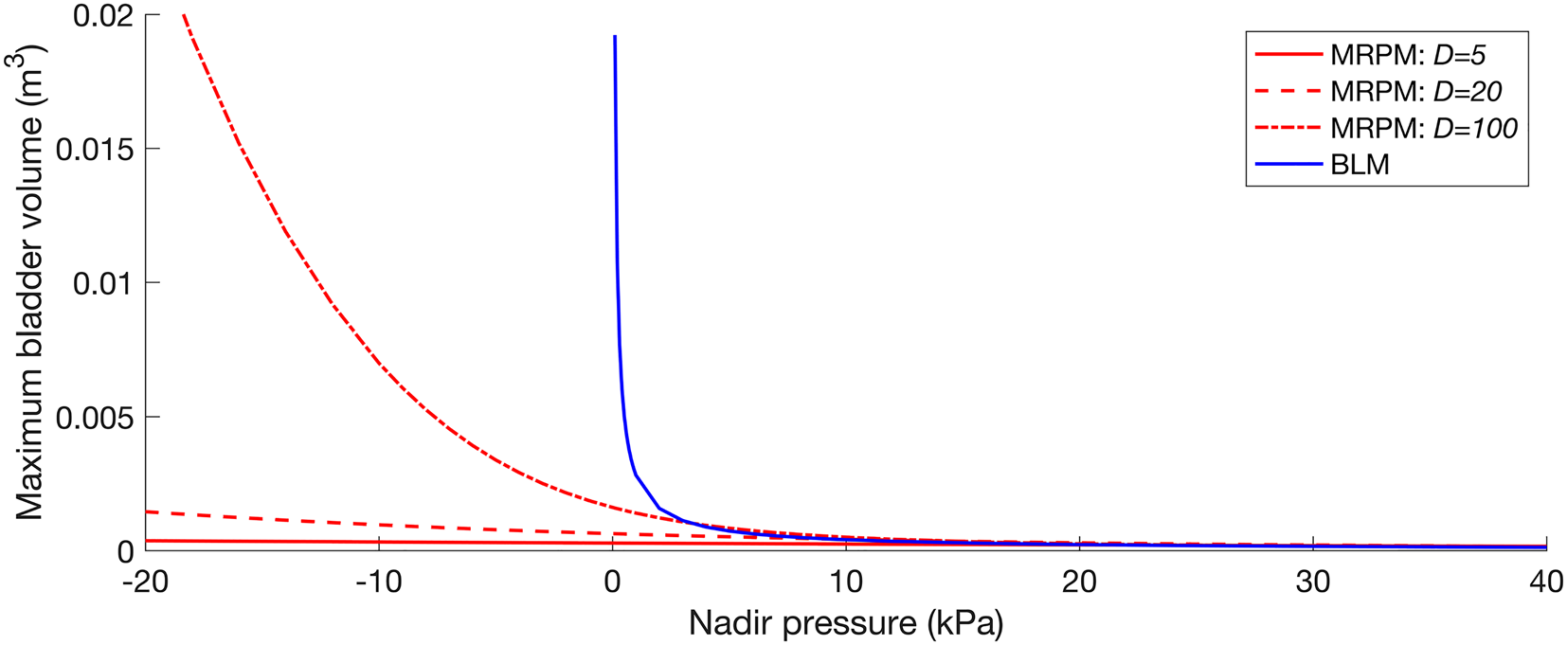
Maximum swim bladder volume (y axis) predicted by the MRPM (red lines) and the BLM (blue line) for the simulated pressure profiles over a range of nadir pressures (x axis) and durations ($D$ = 5, 20 and 100 ms). Note that the BLM is not influenced by rate of pressure change so only one output (blue line) is shown.

The three potential sources of error produced by the BLM are presented in Fig. 7: (1) the BLM starts to predict unrestricted unphysical growth in swim bladder volume as external pressure approaches 0 kPa, (2) the BLM does not produce physically meaningful outputs for negative pressures, and (3) the BLM fails to account for the effects of rate of pressure change, emphasised by the variation in swim bladder volume predicted by the MRPM but not by the BLM at $D$ = 5, 20, and 100 ms (Fig. 7).

### Empirical pressure profiles

**Figure 8:**
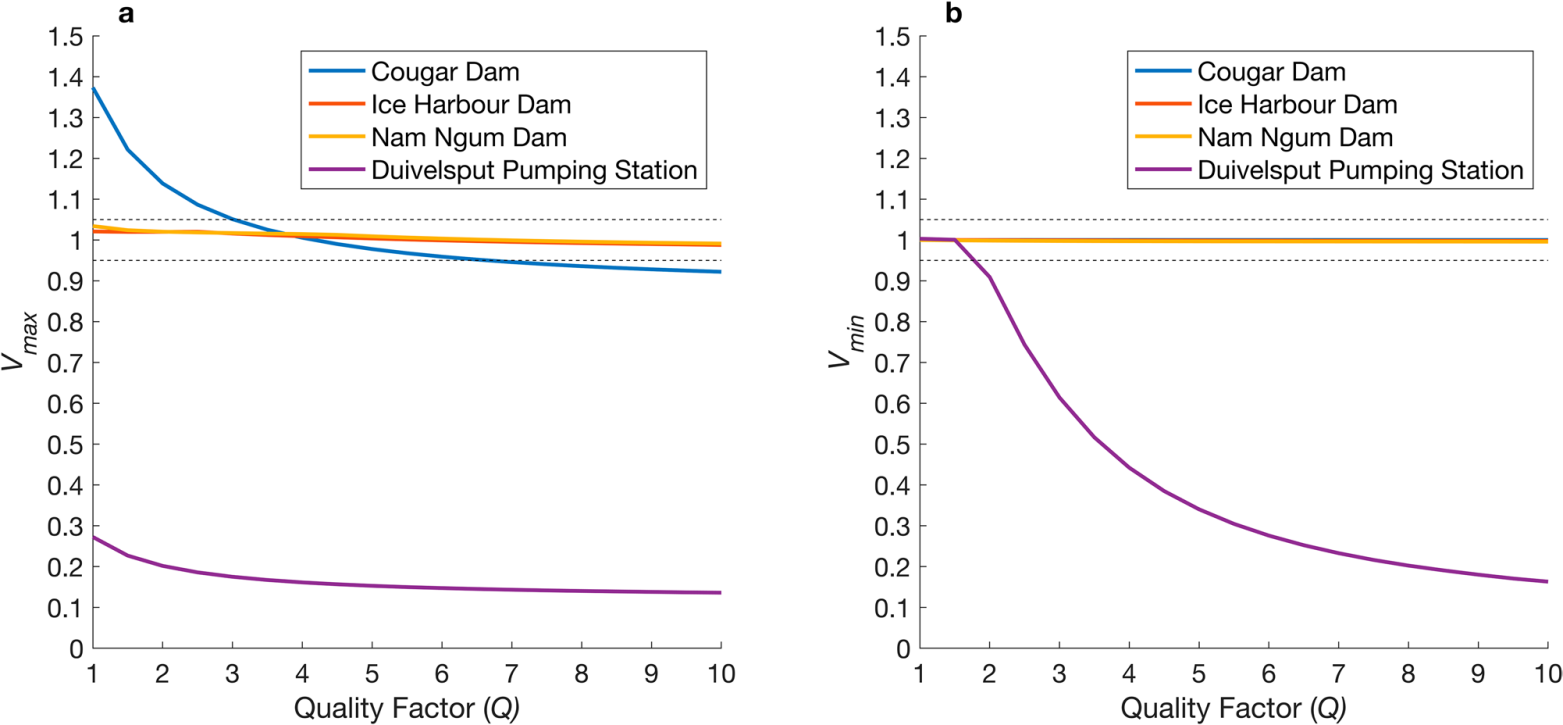
Quotient of the (**a**) maximum (${v}_{\mathrm{max}}$) and (**b**) minimum (${v}_{\mathrm{min}}$) swim bladder volume predicted by the modified Rayleigh Plesset model (MRPM) and Boyle’s Law Model (BLM) using different quality factors (*Q*) for the MRPM. Dashed lines represent the threshold for a 5% difference in predicted volume between swim bladder expansion models.

**Figure 9:**
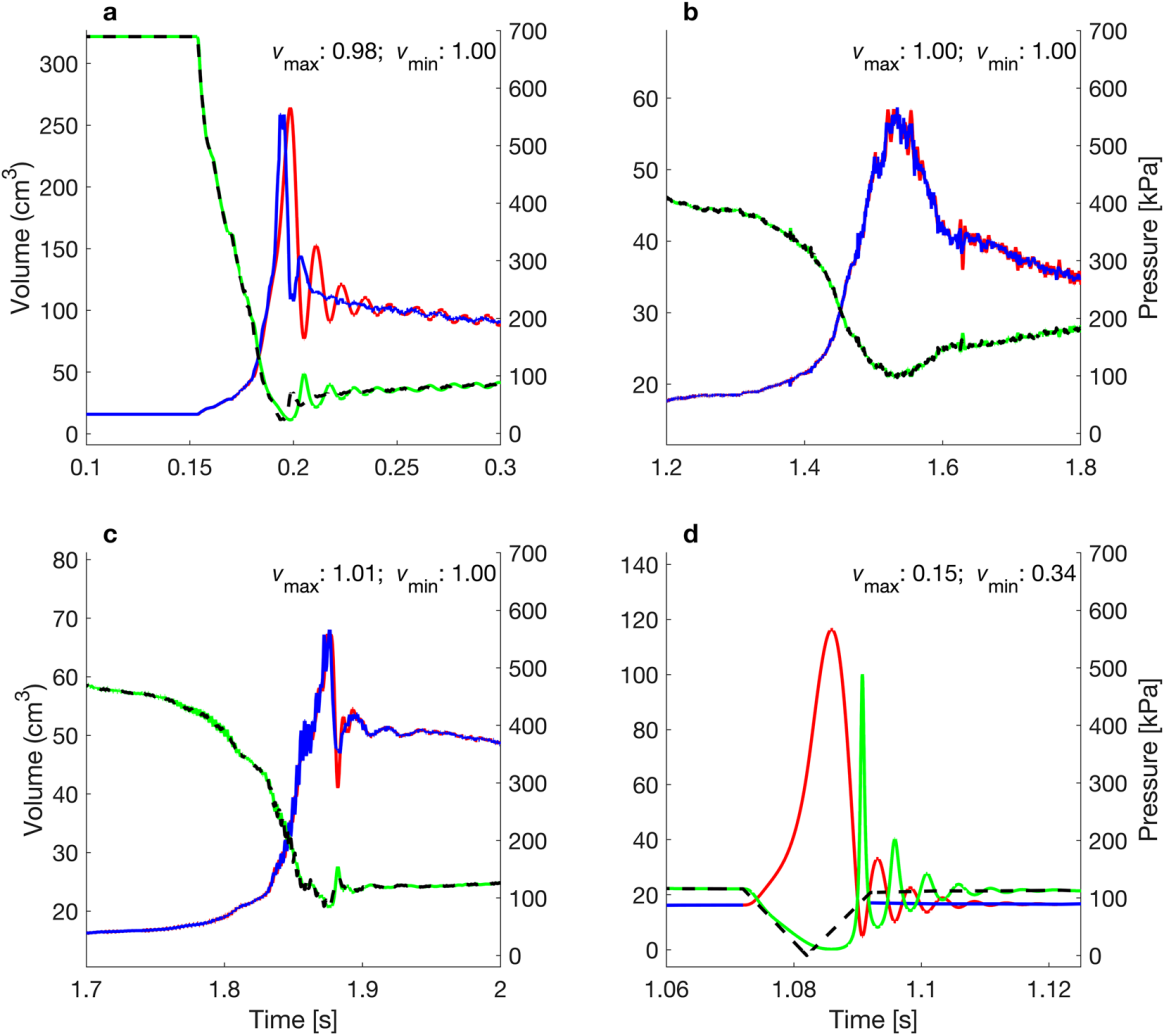
Swim bladder volume (m^3^; left axis) predicted by the BLM (blue line) and the MRPM (red line) for pressure profiles (kPa; right axis; dashed black line) measured with an autonomous sensor at the Cougar (**a**), Ice Harbour (**b**) and Nam Ngum (**c**) Dam and the Duivelsput Pumping Station (**d**). Green line is the internal swim bladder pressure (kPa; right axis) predicted by the MRPM. MRPM data for $Q$ = 5. Quotient of maximum (${v}_{\mathrm{max}}$) and minimum (${v}_{\mathrm{min}}$) swim bladder volume predicted by each model inset in top right of each panel.

At the Cougar Dam, the rate of pressure change was sufficiently rapid that dynamic processes not fully captured by the BLM are likely important. This is highlighted by the BLM overestimating and underestimating maximum swim bladder volume by more than 5% at $Q$ < 3 and $Q$ > 7, respectively (Fig. 8a). At the Ice Harbour and Nam Ngum Dam, ${v}_{\mathrm{max}}$ and ${v}_{\mathrm{min}}$ were approximately one under all values of $Q$, suggesting that rates of pressure change at these locations was slow enough to render dynamic processes unimportant (Figs. 8, 9b,c). At the Duivelsput pumping station, where a brief tension was observed (so small that, within the measurement error, the recorded pressure was 0 Pa), the BLM was unable to predict a swim bladder volume, whereas the MRPM could, resulting in ${v}_{\mathrm{max}}$ and ${v}_{\mathrm{min}}$ values that greatly deviated from one under most $Q$ values (Figs. 8, 9d). At the Cougar Dam and the Duivelsput Pumping Station, large oscillations in swim bladder volume were predicted to occur by the MRPM after rapid decompression as excess kinetic energy was dissipated (Fig. 9a,d). These oscillations occurred concurrently with fluctuations in predicted internal swim bladder pressure, which deviated from the external driving pressure (Fig. 9a,d). At the Duivelsput Pumping Station, at $Q$ = 5, these deviations were large, with the peak in predicted internal pressure that occurred during the first contraction almost reaching 500 kPa, approximately 400 kPa more than the external driving pressure at that time (Fig. 9d).

## Discussion

Swim bladder expansion and rupture due to decompression is a primary cause of injury and mortality in fish passing water infrastructure, such as through turbines and pumping systems^8^. A model that accurately predicts swim bladder size in response to pressure change and subsequent probability of injury and mortality is essential in advancing more sustainable infrastructure design criteria. In this study, two swim bladder expansion models are presented and tested. One based on the quasi-static Boyle’s Law (BLM), conventionally assumed to govern barotrauma in fish, and a more realistic Modified Rayleigh Plesset Model (MRPM) which includes both inertial and pressure functions and is parameterised to represent a fish swim bladder. The two models were tested on a range of (1) simulated and (2) empirically derived pressure profiles. For the simulated pressure profiles, there were a range of conditions that resulted in large differences in the maximum and/or minimum swim bladder size predicted by each model. This is because the BLM fails to incorporate dynamic effects and hence when pressure change occurs rapidly (i.e. proportional to the resonant frequency of the bladder) or when nadir pressure is near or below absolute zero it does not appropriately account for the physical process governing gas volume change. Key examples where such conditions occur at water infrastructure are presented (e.g. the Cougar dam and Duivelsput pumping station), highlighting that this is an applied and not solely theoretical issue. Further work is required to validate the MRPM, and refine input parameters (e.g. the Quality factor), for example to make the result species specific, but it provides a physically more realistic model for predicting swim bladder size when pressure change occurs rapidly and at pressures near or below absolute zero.

The ratio of pressure change (RPC) has been presented as one of the principal variables explaining the occurrence and severity of barotrauma in fish exposed to decompression^6,9,13,41^. Correlations between RPC and most injury types have been identified (e.g. swim bladder rupture, exophthalmia, emboli and haemorrhage) for a range of species^11,12,41^. This is logical as when pressure change occurs slowly (i.e. slower than the resonant frequency of the swim bladder) unsaturated gasses within the fish change volume in proportion to the RPC (i.e. as predicted by Boyle’s Law). However, the results of this study show that, under a range of conditions, some of which occur in situ (e.g. Cougar dam and Duivelsput pumping station), dynamic factors will likely influence how the swim bladder responds to pressure change and as such, are likely important in governing the probability and severity of barotrauma. In line with these findings, researchers should be cautious when using RPC to predict barotrauma injury when rate of pressure change is very fast or if nadir pressures are near or below absolute zero (e.g. close to turbine blades).

The rate of pressure change has also been identified as an influencing factor in barotrauma, although the casual link between rate of change and injury are often unclear. Beirão et al.^11^ found that the period of decompression influenced the occurrence and magnitude of internal haemorrhaging and emboli in pictus catfish (*Pimelodus pictus*). In addition, Brown et al.^9^ demonstrated that rapid decompression, occurring over a few seconds, was more likely to cause severe barotrauma in Chinook salmon (*Oncorhynchus tshawytscha*) than sustained decompression (over a minute). Critically, the studies undertaken by Brown et al.^9^ and Beirão et al.^11^ replicated rates of pressure change that were sufficiently slow that the response of swim bladder volume was likely governed primarily by Boyles’ Law (i.e. outside of the inertial controlled range). Hence, correlations between rate of pressure change and injury are likely due to non-inertial related processes like the ability of an organism to passively or actively mitigate for increasing swim bladder volume. For example, physostomous fish have a pneumatic duct that connects the oesophagus to the swim bladder, and if decompression occurs relatively slowly then they can vent gas, potentially reducing the probability of injury^6^. Importantly, in this study we identify that above a certain threshold the rate of pressure change may also directly influence the probability and severity of barotrauma by causing the swim bladder to expand to a maximum size greater than its equilibrium state. This threshold is proportional to the resonant frequency/period of the swim bladder which varies depending on its size and physical properties and hence will differ between individuals and species. The resonant frequency and period of a swim bladder with radius of 15.6 mm (as used in this study), using best estimates of surface tension and shear viscosity, is *ca.* 200 Hz and 5 ms, respectively. Future research should focus on resolving a conservative threshold rate of pressure change, valid for a wide range of species, at which dynamic processes start to increase barotrauma risk.

In this study, pressure profiles from four water infrastructure sites around the world were presented and the difference in swim bladder maximum or minimum size predicted by the MRPM and BLM assessed. Predictions differed by more than 5% between the two models for two of the case studies, the Cougar dam and Duivelsput pumping station. At the Cougar dam, predicted maximum swim bladder volume deviated between the two models by more than 5% at Quality factors of less than three and more than seven. At the Duivelsput pumping station, the predicted maximum and minimum swim bladder size differed greatly under almost all Quality factors tested. The differences between the two models at the Cougar dam was the result of a rapid rate of decompression that was sufficiently fast to ensure that dynamic processes had a large effect (e.g. a 37% difference in predicted maximum volume at $Q$ = 1). The difference at the Duivelsput pumping station was the result of very small negative absolute nadir pressure for which Boyle’s Law cannot produce a valid estimate of swim bladder size. At both locations, the MRPM also predicted large amplitude oscillations in swim bladder size post decompression and it is possible that such repeated exposure to physical stress could lead to tissue weakening and increase the risk of injury (e.g. as in humans^42^). The MRPM predicted that internal swim bladder pressure increased during the contraction phase of each oscillation. The predicted deviation between the internal swim bladder pressure and external driving pressure was large under certain conditions (e.g. *ca.* 400 kPa at the Duivelsput pumping station at $Q$ = 5). It is possible that this pressure difference could cause pain (e.g. trapped gas in humans^43^) and may put considerable additional strain on internal tissues.

The results of this study clearly indicate that dynamic processes should be considered when assessing swim bladder expansion and contraction if there is any chance that pressure change occurs rapidly (i.e. proportional to the resonant frequency of the bladder), and/or the nadir pressures are low (i.e. at or near tension). However, there is currently insufficient evidence to infer the biologically significance (i.e. individual fitness cost) of the predicted differences in swim bladder response to pressure change between the two models identified in this study. This is because previous studies have typically focussed on correlating exposure to rapid pressure change directly with post-exposure incidence of barotrauma injury^8,10–12^ and it is currently unclear what degree of expansion is required to cause swim bladder rupture. If rupture occurs after only a relatively small amount of expansion, then this might render some of the peak differences in maximum swim bladder size predicted in this study moot. It is recommended that in situ real-time observation of swim bladder size, rupture and resultant secondary injury be undertaken as a research priority, to better understand the mechanisms governing barotrauma and to provide data to validate swim bladder expansion models (i.e. the MRPM).

The empirical profiles presented in this study were selected for having characteristics likely to cause differences between the two models as a proof of concept, hence they are not a representative sample of multiple different types of water infrastructure. The rate of pressure change that occurs at different hydropower dams varies considerably and is governed by a range of complex factors including head difference, time to pass the turbine runners, operating conditions (e.g. discharge) and turbine type^22^. The Cougar dam has a head difference of 138 m and has five Francis turbines with an output ranging from 17.5 to 40 MW. Worldwide, there are many larger dams and many with Francis turbines, so it is not unreasonable to assume that equivalent or faster rates of pressure change could occur elsewhere. Negative absolute pressures occurring at water infrastructure tend to be focussed near the pump or turbine blades^23,24^, often leading to cavitation which can damage the blades over time^44^. However, the size and location of these low-pressure regions varies in space and time and a fish’s risk of exposure to such conditions is dependent on the path it takes through the infrastructure^45^. The very lowest nadir pressures tend to occur in isolated regions near the blade tips and exposure risk to these pressures tends to be low^45^. This low exposure risk might explain why it was only possible to source one empirical pressure profile that recorded tension (i.e. the Duivelsput pumping station) for use in this study. Further work is needed to quantify the exposure risk of fish to nadir pressures near or below absolute zero, at a range of facilities, especially when turbines or pumps are operating sub-optimally which increases the risk of negative pressures occurring^44^.

The Rayleigh Plesset equation, which forms the basis of the MRPM in this study, is able to predict dynamic changes in the radius of a spherical gas bubble surrounded by an infinite body of water exposed to pressure change. However, a fish’s swim bladder is surrounded by flesh, not water. In the MRPM, additional damping, mimicking that caused by flesh surrounding the swim bladder, was artificially incorporated by enhancing the viscosity term and by using an appropriate surface tension value in the Rayleigh Plesset equation. Alternative techniques could also be used to refine model predictions. Leighton^46^ provided an analogous form of the Rayleigh Plesset equation for bubbles in an infinite body of marine mud, and this could readily be adapted to represent a fish swim bladder if key material parameters, such as the shear modulus, shear viscosity and the density of the tissue surrounding the swim bladder, are known. Leighton’s^46^ formulation incorporated viscous losses only, though he indicated how acoustic radiation losses could be included, which has since been achieved^47–49^. Since these models were created to simulate spherical bubbles in marine sediment, if adapted to predict swim bladder dynamics they would assume that the flesh outside the swim bladder extends out to infinity. To resolve this, the Rayleigh Plesset equation could be replaced by a dynamic model of a spherical swim bladder surrounded by a spherical layer of flesh, produced by adapting a model that has been formulated for echo-contrast agents^50,51^. The material properties of the tissues surrounding the swim bladder of a range of fish species at risk of passage through water infrastructure would be required to enable these more advanced models to be formulated. While the MRPM used in this study does not directly account for the effects of the surrounding tissue, they are artificially represented by enhancing the viscosity term and using a range of appropriate Quality factors ($Q$) reported in the literature. Hence, although model validation and refinement are needed, the MRPM provides a good insight as to whether dynamic processes are important in governing swim bladder size in response to pressure change. Suggested model refinements include improving parameter estimates for the polytropic index ($k$) and vapour pressure (${p}_{v}$) used in the MRPM. Both of which could affect predicted swim bladder size but the effects of which were not assessed in this study, to focus on the influence of dynamic processes.

The results of this study suggests that a model based on Boyle’s Law is inappropriate for predicting swim bladder size under a range of conditions that fish may experience as they pass through turbines and pumps due to the influence of dynamic processes. The prevalence of such conditions at the diverse array of infrastructure present in freshwater ecosystems worldwide is currently unknown, but they do occur, i.e. at the Cougar Dam, USA, and Duivelsput pumping station, Belgium, emphasising that this is an applied not just theoretical issue. The alternate model presented, the MRPM, is free of the quasi-static and equilibrium assumptions that render Boyle’s Law inappropriate for modelling the response of the swim bladder to tensions and the effects of resonances, inertia and damping. Consequently, the MRPM is more appropriate for predicting swim bladder size in response to pressure change, as it produces physically realistic results under a greater range of conditions than Boyle’s Law. Further refinement may be needed but it is a more realistic base model for inferring barotrauma in fish that pass through turbines and pumps and a more appropriate option to inform the long-term development of sustainable water infrastructure.

### Supplementary Information

Supplementary Information.

Supplementary Figures.

## Data Availability

All data supporting this study are openly available from the University of Southampton repository at 10.5258/SOTON/D2845.
