# Supplementary material for: Boyle’s Law ignores dynamic processes in governing barotrauma in fish
Source: Sci Rep. 2023 Nov 5;13:19125. doi: 10.1038/s41598-023-46125-9 (PMC10625976; doi:10.1038/s41598-023-46125-9)
Supplement: Supplementary file 1 — Supplementary Information. [file 41598_2023_46125_MOESM1_ESM.pdf]

Relating Damping in the Rayleigh Plesset Equation to the Quality Factor

(a) Linearising the Rayleigh-Plesset equation:

The terms in the Rayleigh Plesset equation (RPE), Eq 3 in the paper, can be rearranged as follows:

$$R\ddot{R}+\frac{3}{2}\dot{R}^2=\frac{1}{\rho}\left\{\left(P_0+\frac{2\sigma}{R_0}-p_v\right)\left(\frac{R_0}{R}\right)^{3\kappa}-\left(P_0+\frac{2\sigma}{R}-p_v\right)-\frac{4\eta\dot{R}}{R}-P(t)\right\}$$

$$\ddot{R}=-\frac{3}{2}\frac{\dot{R}^2}{R}+\frac{1}{\rho R}\left(P_0+\frac{2\sigma}{R_0}-p_v\right)\left(\frac{R_0}{R}\right)^{3\kappa}-\left(P_0+\frac{2\sigma}{R}-p_v\right)\frac{1}{\rho R}-\frac{4\eta\dot{R}}{\rho R^2}-\frac{P(t)}{\rho R} \quad \text{(S1)}$$

$$\ddot{R}=-\frac{3}{2}\frac{\dot{R}^2}{R}+\frac{1}{\rho}\left(P_0+\frac{2\sigma}{R_0}-p_v\right)R_0^{3\kappa}\frac{1}{R^{3\kappa+1}}-\frac{(P_0-p_v)}{\rho}\frac{1}{R}-\frac{2\sigma}{\rho}\frac{1}{R^2}-\frac{4\eta\dot{R}}{\rho R^2}-\frac{P(t)}{\rho R}$$

Writing the RPE using constants, a_1-a_5, to simplify the following expressions, gives:

$$\ddot{R}=a_1\frac{\dot{R}^2}{R}+a_2\frac{1}{R}+a_3\frac{1}{R^2}+a_4\frac{1}{R^{3\kappa+1}}+a_5\frac{\dot{R}}{R^2}-\frac{p(t)}{\rho R} \quad \text{(S2)}$$

$$a_1=-\frac{3}{2},\quad a_2=-\frac{(P_0-p_v)}{\rho},\quad a_3=-\frac{2\sigma}{\rho},\quad a_4=\left(P_0+\frac{2\sigma}{R_0}-p_v\right)\left(\frac{R_0^{3\kappa}}{\rho}\right),\quad a_5=-\frac{4\eta}{\rho} \quad \text{(S3)}$$

Now to express the RPE in state-space form, choosing the states as:

$$x_1=R$$
$$x_2=\dot{R} \quad \text{(S4)}$$

To form the state vector:

$$\underline{x}=\begin{bmatrix}x_1\\x_2\end{bmatrix} \quad \text{(S5)}$$

The input, or driving pressure, can be expressed as:

$$\underline{u}=\begin{bmatrix}0\\u(t)\end{bmatrix}=\begin{bmatrix}0\\-P(t)/\rho R\end{bmatrix}=\begin{bmatrix}0\\-P(t)/\rho x_1\end{bmatrix} \quad \text{(S6)}$$

which can be used to construct the following state-space representation of the RPEs:

$$\dot{\underline{x}}=\begin{bmatrix}\dot{x}_1\\\dot{x}_2\end{bmatrix}=\begin{bmatrix}\dot{R}\\\ddot{R}\end{bmatrix}=\begin{bmatrix}x_2\\a_1\frac{x_2^{\,2}}{x_1}+a_2\frac{1}{x_1}+a_3\frac{1}{x_1^{\,2}}+a_4\frac{1}{x_1^{\,3\kappa+1}}+a_5\frac{x_2}{x_1^{\,2}}\end{bmatrix}+\begin{bmatrix}0\\-\frac{p(t)}{\rho x_1}\end{bmatrix}=\begin{bmatrix}f_1(\underline{x})\\f_2(\underline{x})\end{bmatrix}+\underline{u}(t) \quad \text{(S7)}$$

Linearising the state space model, using the Jacobian, about the equilibrium $x_1=R_0, x_2=0$, gives:

$$\underline{\dot{x}} = A\underline{x} + \underline{u}(t)$$

$$A = \begin{bmatrix} \frac{\partial f_1}{\partial x_1} & \frac{\partial f_1}{\partial x_2} \\ \frac{\partial f_2}{\partial x_1} & \frac{\partial f_2}{\partial x_2} \end{bmatrix} = \begin{bmatrix} 0 & 1 \\ -a_1 \frac{x_2^2}{x_1^2} - a_2 \frac{1}{x_1^2} - a_3 \frac{2}{x_1^3} - a_4 \frac{(3\kappa+1)}{x_1^{3\kappa+2}} - a_5 \frac{2x_2}{x_1^3} & 2a_1 \frac{x_2}{x_1} + a_5 \frac{1}{x_1^2} \end{bmatrix} \quad \text{(S8)}$$

Substituting $x_1 = R_0, x_2 = 0$, the equilibrium point about which the linearisation is formed, gives:

$$\underline{\dot{x}} = \begin{bmatrix} 0 & 1 \\ -a_2 \frac{1}{R_0^2} - a_3 \frac{2}{R_0^3} - a_4 \frac{(3\kappa+1)}{R_0^{3\kappa+2}} & a_5 \frac{1}{R_0^2} \end{bmatrix} \underline{x} + \underline{u}(t)$$

$$\Rightarrow \ddot{R} - \frac{a_5}{R_0^2}\dot{R} + \left(a_2 \frac{1}{R_0^2} + a_3 \frac{2}{R_0^3} + a_4 \frac{(3\kappa+1)}{R_0^{3\kappa+2}} \right) R = -\frac{p(t)}{\rho R_0} = u(t) \quad \text{(S9)}$$

Using the definitions of the constants (a_k), gives:

$$\ddot{R} + \frac{4\eta}{\rho R_0^2}\dot{R} + \left(\left(P_0 + \frac{2\sigma}{R_0} - p_v \right) \frac{(3\kappa+1)}{\rho R_0^2} - \frac{(P_0 - p_v)}{\rho} \frac{1}{R_0^2} - \frac{4\sigma}{R_0^3 \rho} \right) R = u(t) \quad \text{(S10)}$$

(b) Finding the oscillatory frequency in conditions of ligh damping

Common parlance gives numerous ways to define the resonance frequency of a bubble, depending on how the motion is damped, whether the motion is returning to equilibrium following some displacing force that has now ceased, or whether the system is responding to some oscillatory driving force and we are defining resonance as occurring at the frequency of the driving signal that gives us most scattered pressure, greatest bubble wall displacement, greatest bubble wall speed, and many other factors. These give subtly different expressions for the 'bubble resonance' (Ainslie and Leighton, 2011).

Here wecompare Equation (S10) to the equation for a unforced mass-spring-damper system (Equation (3.151) of Leighton, 1994) by setting *u*(*t*)=0, i.e. considering the time after which an initial excitation has ceased:

$$\ddot{R} + 2\beta\dot{R} + \omega_0^2 R = u(t) = 0 \quad \text{(S11)}$$

Here, ω_0 is the undamped natural frequency, and Q is the quality factor, whichis defined as $Q = \frac{\omega_b}{2\beta}$ where $\omega_b^2 = \omega_0^2 - \beta^2$ is the circular frequency at which the bubble oscillates under conditions of light damping (defined as occurring when $\beta < \omega_0$).. Equating terms for the linearised RP model (equation (S10)) with *u*(*t*) set to zero (as discussed above), with equation (S11), we can see.

$$\omega_0 = \sqrt{\left(\left(P_0 + \frac{2\sigma}{R_0} - p_v \right) \frac{(3\kappa+1)}{\rho R_0^2} - \frac{(P_0 - p_v)}{\rho R_0^2} - \frac{4\sigma}{R_0^3 \rho} \right)} = \frac{1}{\sqrt{\rho} R_0} \sqrt{\left(\left(P_0 + \frac{2\sigma}{R_0} - p_v \right)(3\kappa+1) - (P_0 - p_v) - \frac{4\sigma}{R_0} \right)}$$

$$= \frac{1}{\sqrt{\rho} R_0} \sqrt{3\kappa(P_0 - p_v) + \left(\frac{2\sigma}{R_0} \right)(3\kappa - 1)}$$

(S12)

$$2\beta = \frac{4\eta}{\rho R_0^{\ 2}} \tag{S13}$$

To compute the frequency of damped oscillations after the initial forcing has ceased (ω_b):

$$\omega_b^{\ 2} = \omega_0^{\ 2} - \beta^2 = \frac{1}{\rho R_0^{\ 2}}\left(3\kappa\left(P_0 - p_v\right) + \left(\frac{2\sigma}{R_0}\right)\left(3\kappa - 1\right)\right) - \frac{4\eta^2}{\rho^2 R_0^{\ 4}} = \frac{1}{\rho R_0^{\ 2}}\left(3\kappa\left(P_0 - p_v\right) + \left(\frac{2\sigma}{R_0}\right)\left(3\kappa - 1\right) - \frac{4\eta^2}{\rho R_0^{\ 2}}\right)$$

(S14)

$$\omega_b = \sqrt{\omega_0^{\ 2} - \beta^2} = \frac{1}{\sqrt{\rho}R_0}\sqrt{3\kappa\left(P_0 - p_v\right) + \left(\frac{2\sigma}{R_0}\right)\left(3\kappa - 1\right) - \frac{4\eta^2}{\rho R_0^{\ 2}}} \tag{S15}$$

which can be combined with the loss factor to compute $Q = \frac{\omega_b}{2\beta}$.

$$Q = \frac{\sqrt{\rho}R_0\sqrt{3\kappa\left(P_0 - p_v\right) + \left(\frac{2\sigma}{R_0}\right)\left(3\kappa - 1\right) - \frac{4\eta^2}{\rho R_0^{\ 2}}}}{4\eta} \tag{S16}$$

References

Ainslie, M.A. and Leighton, T.G. (2011) Review of scattering and extinction cross-sections, damping factors, and resonance frequencies of a spherical gas bubble, Journal of the Acoustical Society of America, 130(5), 3184-3208 (doi: 10.1121/1.3628321).

Leighton, T.G. (1994) The Acoustic Bubble, Academic Press, 640 pages (ISBN 0124419208).
