# Supplementary material for: Boyle’s Law ignores dynamic processes in governing barotrauma in fish
Source: Sci Rep. 2023 Nov 5;13:19125. doi: 10.1038/s41598-023-46125-9 (PMC10625976; doi:10.1038/s41598-023-46125-9)
Supplement: Supplementary file 2 — Supplementary Figures. [file 41598_2023_46125_MOESM2_ESM.pdf]

Supplementary figures

Manuscript: Boyle's Law ignores dynamic processes in governing barotrauma in fish.

Authors: Kerr, J.R, White, P.R., Leighton, T.G., Silva, L.G.M. and Kemp, P.S.

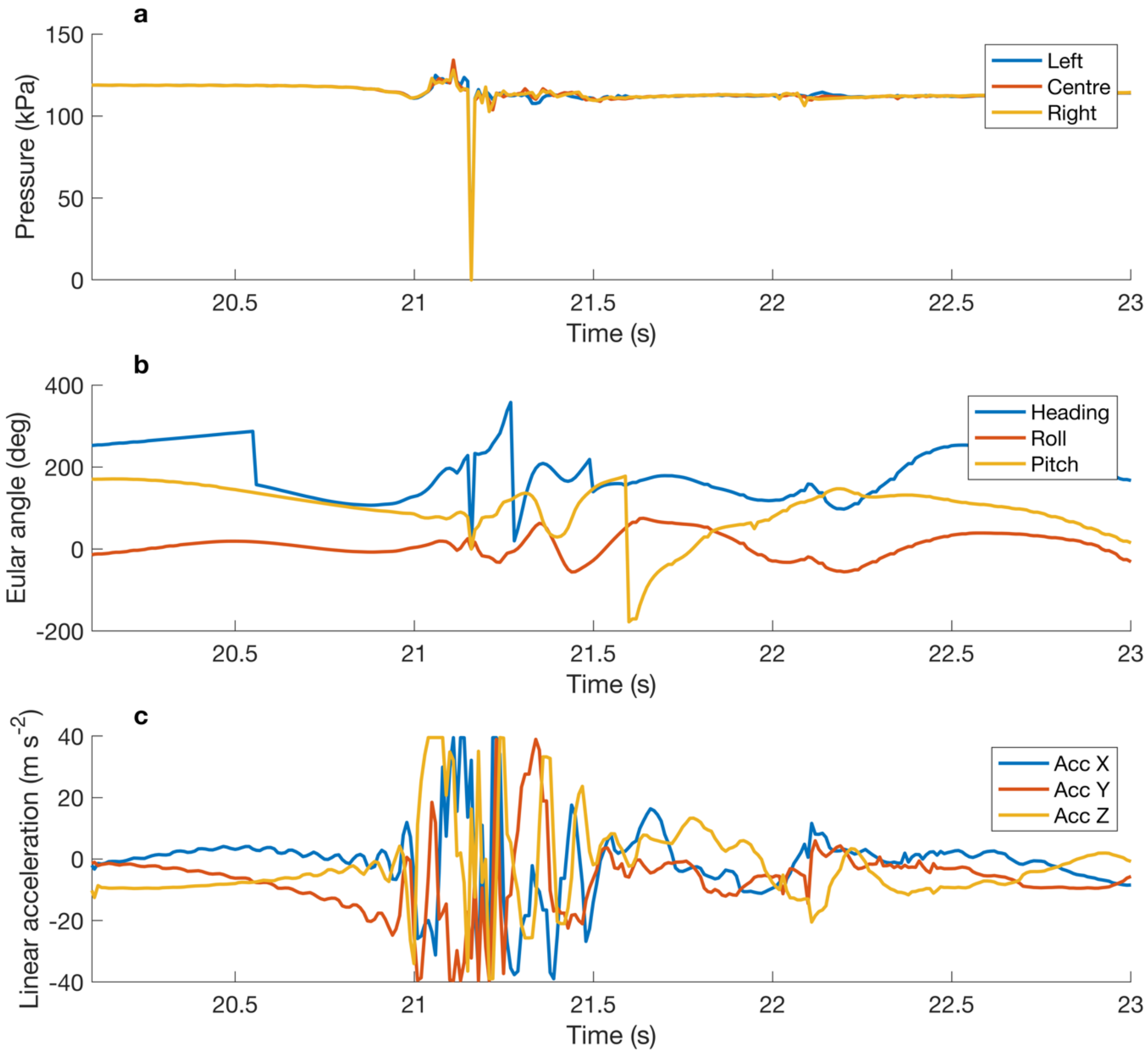

Figure S1. Probe data from Trial B281212134647 at the Duivelsput pumping station. **a**) is pressure (kPa) from the left, centre, and right pressure sensor. **b**) is Euler angle (degrees) for the heading, pitch, and roll. **c**) is the linear acceleration (m s^{-2}) in X, Y, and Z.

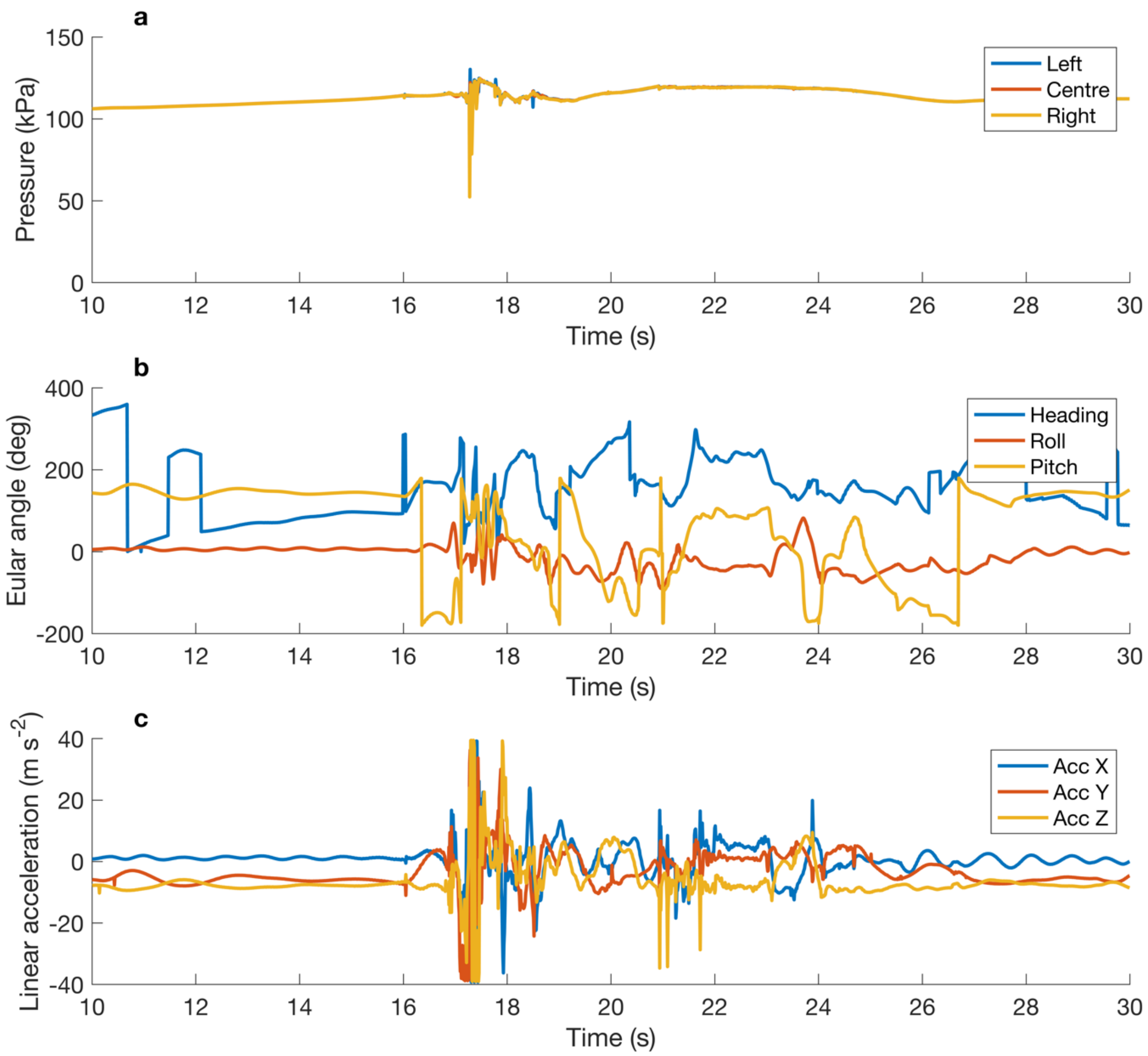

Figure S2. Probe data from Trial B271212134548 at the Duivelsput pumping station. **a**) is pressure (kPa) from the left, centre, and right pressure sensor. **b**) is Euler angle (degrees) for the heading, pitch, and roll. **c**) is the linear acceleration (m s^{-2}) in X, Y, and Z.
